# On Scaling of Scientific Knowledge Production in U.S. Metropolitan Areas

**DOI:** 10.1371/journal.pone.0110805

**Published:** 2014-10-29

**Authors:** Önder Nomaler, Koen Frenken, Gaston Heimeriks

**Affiliations:** 1 School of Innovation Sciences, Eindhoven University of Technology, Eindhoven, The Netherlands; 2 Copernicus Institute of Sustainable Development, Utrecht University, Utrecht, The Netherlands; Katholieke Universiteit Leuven, Belgium

## Abstract

Using data on all scientific publications from the Scopus database, we find a superlinear scaling effect for U.S. metropolitan areas as indicated by the increase of per capita publication output with city size. We also find that the variance of residuals is much higher for mid-sized cities (100,000 to 500,000 inhabitants) compared to larger cities. The latter result is indicative of the critical mass required to establish a scientific center in a particular discipline. Finally, we observe that the largest cities publish much less than the scaling law would predict, indicating that the largest cities are relatively unattractive locations for scientific research.

## Introduction

Many phenomena scale with city size and do this in a non-linear way. Bettencourt et al. (2010) find that as city size increases, per capita quantities such as wages and GDP increase by approximately 15% more than the expected linear growth. Elsewhere, it was established that gross domestic expenditure on R&D (GERD) & gross domestic product (GDP) and GDP & POP (population) all exhibit strong scaling effects [1]. However, also undesired properties tend to increase following the same 15% rule, including crime, traffic congestion and certain diseases [2].

For knowledge production activities, scaling effects have been found to be even bigger. Depending on the indicator, Bettencourt et al. [3] found scaling percentages from 25 percent for inventors, 27 percent for patents, and 34 percent for R&D employment. Following these striking empirical regularities, indicated as “scaling laws”, it is tempting to benchmark individual cities according to the “expected performance” given their city size. Indeed, it has been argued that scaling laws can provide a building block for “a new science of performance-based planning” [2].

We show below that such an approach does not apply well to the production of scientific knowledge. Indeed, data on scientific output of U.S. cities show that the per capita number of scientific papers increases with city size and the exponent is exceptionally high. However, the statistical regularity is weak as many mid-sized cities publish much less than would be predicted by the scaling law, while for some other (exceptional) “science cities,” publishing performance is much higher than would be predicted from their modest size.

We also observe that the very large cities publish much less than the scaling law would predict, indicative of agglomeration disadvantages for very large cities. We interpret this result as evidence that, relatively, very large cities are unattractive locations for scientific research. This effect may well be due to high rental prices that both universities themselves, as well as their staff and students have to pay for being located in such very large cities.

A disaggregated analysis at the level of scientific discipline further shows large disciplinary differences in the scaling behavior as well as in the goodness of fit of the alleged “scaling law”. In particular, we find that scaling applies best to disciplines with a strong local interaction with citizens or firms (such as medical and engineering sciences). We understand this result as reflecting that agglomeration advantages are most apparent in scientific research which is closely linked to a local user base.

## Scaling

Bettencourt et al. [2] argue that superlinear scaling of social outputs is related to the number of possible social interactions at a local scale that increases exponentially with city size. This magnifies the effects of spatially-delimited social interaction to create even greater levels of social output, whether positive (such as wealth and innovation) or negative (e.g., crime and poverty).

In spatial economics, scaling patterns are often explained as productivity gains that result from economies of scale, the mobility of labor, knowledge spillovers, and other effects of agglomeration economies [4], [5]. Similarly, concentration of research is also explained in terms of agglomeration advantages in spatial scientometrics [6].

Agglomeration advantages in research are efficiency gains for a researcher or research institute stemming from co-locating in a geographical cluster, that is, in the vicinity of many other researchers or research institutes, respectively. Advantages stem primarily from cost advantages in search costs for partners and staff, sharing of infrastructure, and the availability of supporting services. Furthermore, the cost of collaboration is lower as travel costs increase with physical distance [6].

A scaling law describes one quantity (*Y*) as a function of the size of another quantity (*N*), such that:
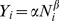
(1)Here, we look at the scaling of scientific output of city *i* (*Y_i_*) as a function of the population of city *i*.

Equation (1) is equivalent to:
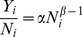
(2)in per capita terms. The exponent *β* indicates the extent of scaling. For *β = 1*, we have a simple linear relation indicating that per capita scientific output is constant over city size (For example, as found for retail [7] and CO_2_ emissions [8]).

For *β>1*, we would have a superlinear relation with per capita scientific output increasing with city size, while for *β<1*, we would have a sublinear relation with per capita scientific output decreasing with city size.

Empirically, using a double log-transformation, we can estimate equation (1) as:
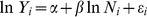
(3)using Ordinary Least Squares (OLS) with *ε* as Gaussian white noise. Looking at urban scaling, one usually takes for *N* the urban population as the indicator of urban size [3], [7]. Here, *Y* stands for urban scientific output measured by the number of published scientific papers.

## Data and Methods

Our geographical unit of analysis is a metropolitan and micropolitan statistical area. These areas, also known as the **C**ore **B**ased **S**tatistical **A**reas (CBSA) collectively, are defined by the US Office of Management and Budget (OMB). Each area is uniquely referred to by a code called as the CBSA code. As the name implies, a CBSA is a group of adjacent areas that are socioeconomically close to an urban center. Note that, in this paper we accordingly use the terms “CBSA” and “city” almost interchangeably for mere convention. Accordingly we built a dataset that has 923 observations, each for another CBSA.

Using an off-line version of Elsevier’s Scopus database covering the time period 1996–2008, we extracted all (about 4.4 million) documents that have at least one author whose (at least one) affiliation reports an address in the US. Together with the delineation tables (version 2003) made available by the US Census Bureau that maps the association between city/town names and the CBSA codes, we were able to standardize *95.25*% of the approximately 8.11 million addresses found in Scopus. On the basis of fractional accounting this is equivalent to 93.1% of all documents.

For each CBSA, we use the US 2000 Census for urban population *N*. Total number of publications in a city is derived from Scopus for the period 1996–2008, which reports author affiliation in the respective area. We use fractional counting meaning that a city occurring on a paper with *x* affiliations is counted only as *1/x*. The total number of publications is further broken down to scientific disciplines according to the classification by Scopus at the 2 digit level (thus 27 disciplines). Publications that report multiple scientific disciplines are accounted for fractionally as well.

Methodically, the primary research question posed by this paper would not require anything more than running the aggregate-level regression specified by equation 2, along with its 27 counterparts at the individual 2-digit discipline level, using equation (3). However, the error terms of all these regressions are systematically heteroskedastic in a peculiar (i.e., inverted U-shaped) way, which, for this paper, is not only a technical issue to deal with, but a major point to make. This pattern of heteroskedasticity reflects that the scaling law explains rather well the scientific output of small or very large cities, while the output of mid-sized cities is not well explained. Given the inverted U-shape of the error term when plotted against population, one can also characterize scientific disciplines in terms of the city size at which the variation unexplained by scaling laws is maximized.

To deal with the problem of heteroskedasticity, we apply a weighting scheme such that the observations of scientific output of medium-sized cities are weighted more according to a function that estimates the error term as an inversely U-shaped function of urban population (see Text S1). Below, we report the estimated coefficients (*α* and *β*) of both the non-weighted (thus heteroskedastic) and the weighted regressions.

## Results for All Disciplines

**Figure 1:**
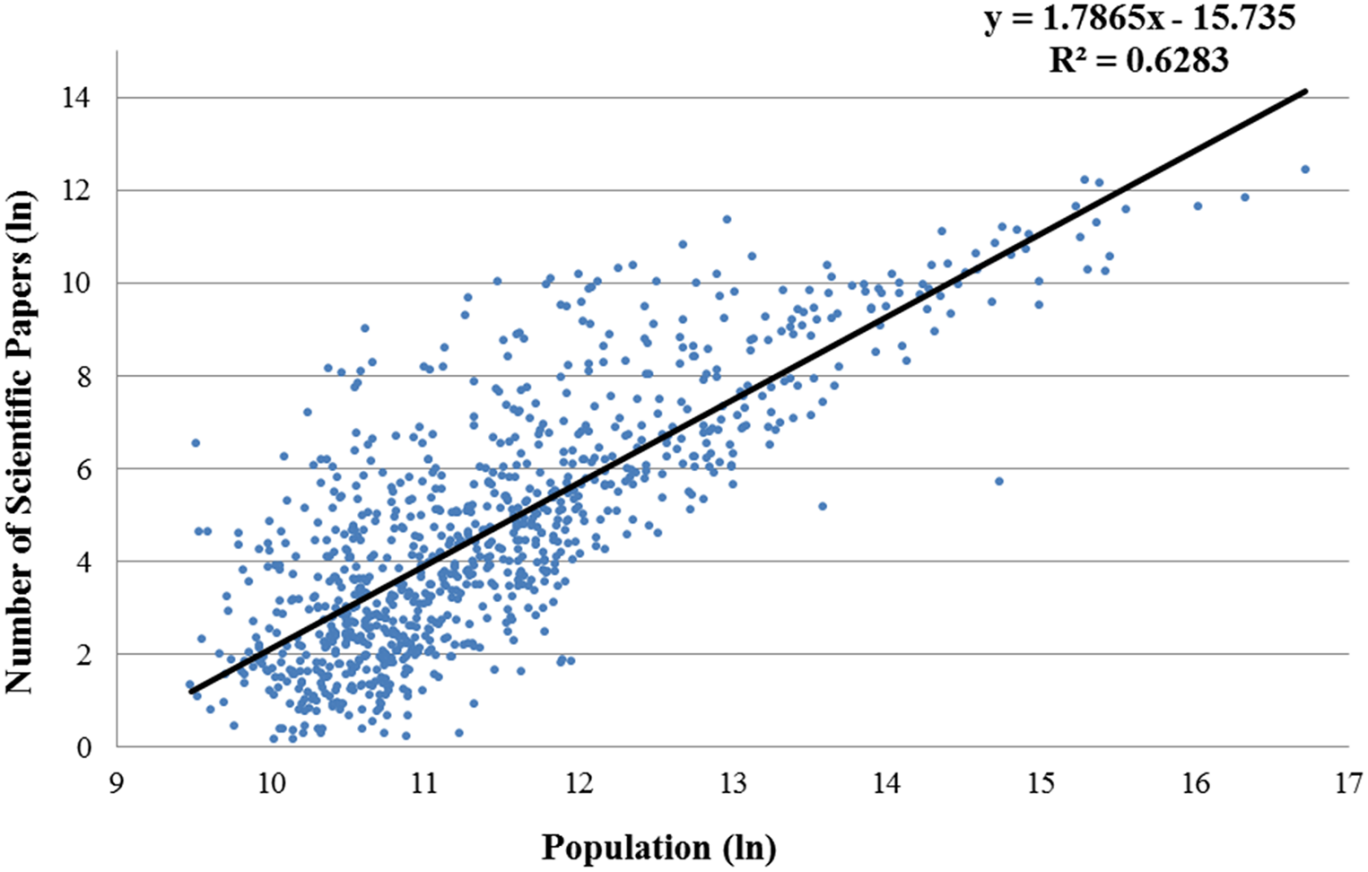
Urban scaling of scientific articles (all disciplines), non-weighted regression.

Figure 1 plots (in log-log scale) the number of scientific publications in all disciplines for each U.S. metropolitan area of different population size. The OLS regression line described the scaling law as specified in equation (3). The estimated exponent *β = 1.78* of the non-weighted regression (as well as *β = 1.676* of the weighted one) clearly lies above 1 and is indicative of a superlinear relationship meaning that the per capita scientific output increases quickly with urban population.

Interestingly, the value of the exponent is found to be substantially higher than that of R&D employment (*β = 1.34*), patenting (*β = 1.27*) or inventors (*β = 1.25*) in U.S. cities [3]. This suggests that scientific knowledge production is even more clustered in larger cities indicative of strong agglomeration advantage in scientific research. However, it would be unwise to jump to the conclusion that science is a big-city enterprise, let alone that science policy should aim to concentrate science in the largest cities.

First of all, the overrepresentation of science in larger cities does not indicate in itself that scientific knowledge production benefits from locating in larger cities. Observing a superlinear relationship between population and scientific output does not indicate that there are agglomeration advantages (i.e. positive externalities) in doing science in larger cities compared to smaller cities. Scaling laws regarding scientific output have also been observed at the level of countries [9], [10] and universities. In addition, it has been found that scientific articles from larger countries or larger universities receive, on average, more citations than articles from smaller countries or universities [11], [12]. This pattern may point to economies of scale at the country and university level. However, it may also reflect citation bias with authors preferentially citing authors from the same country and university.

Alternative candidate explanations include the preference of staff and students to live in larger cities [13], mobility of productive scientists towards elite institutions over-represented in large cities [14], or a bias in the allocation of public funds towards larger cities (cf. [15]). Agglomeration advantages in scientific research can only be precisely measured by looking at whether the productivity of researchers increases with city size [6], [16]. Hence, from a policy point of view, the scaling law alone does not legitimate *per se* a further concentration of resources in larger cities.

Two other important caveats apply as well. First, it is important to note that even though the scaling exponent is very high, the largest three cities (New York, Los Angeles and Chicago) are much less productive than the scaling law predict. For example, the city of New York (The CBSA New York-Northern New Jersey-Long Island, to be more precise) with about 18 million inhabitants is predicted to produce about 774 thousand papers, while it only produces about 253 thousand. Hence, looking at the total set of U.S. cities, scientific production seems over-represented in larger cities reading from the estimated exponent of 1.676. However, this strong logic of urban scaling clearly no longer applies when looking only at the largest three metropolitan areas. This finding resonates with the historical trend of geographical deconcentration in scientific knowledge production found by Grossetti et al. [17].

Second, the statistical fit of the scaling law is rather weak as indicated by a *R^2^* value of *0.63*. In this, the observed pattern is very different from the high *R^2^* values found for urban scaling laws regarding wages (*R^2^ = 0.96*) and private R&D employment (*R^2^ = 0.92*), and more in line with the lower *R^2^* values found for patents (*R^2^ = 0.72*) and R&D establishments (*R^2^ = 0.76*) [3]. The main reason for the weak statistical fit concerns the high variance in output for mid-sized cities up to a population size of about 500,000 inhabitants (or *13.12* in logarithmic scale as in Figure 1). The variance reflects that most mid-sized cities produce little scientific papers while some produce much more than one expects from the scaling law. This can be understood from the ‘chunky’ nature of the organization of science. Most research is done in universities or large research organization. Hence, the many small cities where such organizations are absent produce few papers, while the few small cities that happen to have such establishment within their boundaries produce much more output that the scaling law would predict. The city size with the highest variance is about 73,500 (or 11.2 in logarithmic scale, as visible in Figure A.1a) inhabitants. Around this city size, most cities produce very little scientific papers, while some “science cities” produce a very substantial number of papers. In this pattern, one can readily recognize the small university cities otherwise referred to as “campus towns”. For example, Ames IA (where Iowa State University is located) produced about 16 thousand papers only with a population about 80 thousand.

To conclude, scientific output scales with city size but this pattern is not very systematic. In particular, there are two systematic deviations. First, the very large cities greatly underperform. Second, small cities tend to produce little science with some notable exceptions.

## Disciplinary Differences

Table 1 provides the estimations for the scaling laws at the level of disciplines following the disciplines codes in Scopus. The results show a great deal of heterogeneity across disciplines.

**Table 1:** Scaling laws for scientific disciplines.

Discipline	Non-Weighted OLS			Weighted OLS		
	α	β	R^2^	α	β	Size at Max Variance
Multidisciplinary	−10.21	1.01	*46.41%*	−9.29	0.94	520,967
Agricultural and Biological Sciences	−10.84	1.2	*45.70%*	−10.23	1.15	107,518
Arts and Humanities	−8.33	0.86	*48.01%*	−8.83	0.94	252,769
Biochemistry, Genetics and Molecular Biology	−15.98	1.6	*56.93%*	−15.02	1.54	197,972
Business, Management and Accounting	−9.76	1.01	*49.81%*	−10.18	1.05	160,787
Chemical Engineering	−10.99	1.1	*51.95%*	–10.83	1.1	227,380
Chemistry	−12.93	1.32	*51.03%*	−12.34	1.28	189,463
Computer Science	−12.52	1.26	*51.08%*	−12.48	1.27	205,001
Decision Sciences	−8.19	0.81	*43.53%*	−8.68	0.87	277,600
Earth and Planetary Sciences	−11.28	1.17	*46.34%*	−10.92	1.15	245,183
Economics, Econometrics and Finance	−9.18	0.95	*42.24%*	−10.01	1.03	210,686
Energy	−9.29	0.93	*46.00%*	−9.21	0.93	315,249
Engineering	−14.6	1.49	*59.71%*	−13.55	1.41	165,703
Environmental Science	–10.91	1.17	*47.40%*	−10.4	1.13	141,001
Immunology and Microbiology	−13.24	1.3	*52.70%*	−12.48	1.25	274,728
Materials Science	−12.65	1.28	*53.74%*	−12.1	1.24	221,451
Mathematics	−10.96	1.15	*44.51%*	−11.21	1.18	179,811
Medicine	−17.31	1.78	*71.50%*	−16.61	1.72	235,464
Neuroscience	−13.92	1.35	*54.00%*	−10.29	1.08	381,182
Nursing	−12.11	1.2	*65.63%*	−10.45	1.07	542,203
Pharmacology, Toxicology and Pharmaceutics	−13.55	1.33	*55.52%*	−12.84	1.29	261,306
Physics and Astronomy	−14.24	1.44	*53.14%*	−13.72	1.41	208,635
Psychology	−12.01	1.24	*52.95%*	−11.75	1.23	171,800
Social Sciences	−12.71	1.32	*56.41%*	−12	1.27	147,754
Veterinary	−8.36	0.84	*41.91%*	−8.32	0.85	248,996
Dentistry	−11.28	1.04	*55.35%*	−7.02	0.69	1,202,604
Health Professions	−12.08	1.18	*62.11%*	−7.3	0.79	613,336
**ALL DISCIPLINES**	**−15.73**	**1.79**	***62.83%***	**−14.47**	**1.68**	**73,466**

Some disciplines show strong signs of scaling, in particular Medicine (*β = 1.72*), Biochemistry, Genetics and Molecular Biology (*β = 1.54*) and Engineering (*β = 1.41*). These disciplines also show the best fits reading from the *R^2^* values (*R^2^ = 0.71*, *R^2^ = 0.57* and *R^2^ = 0.60*, respectively). What these disciplines have in common is that these are applied sciences characterized by intensive interaction with local stakeholders, be it patients or companies. The high beta coefficients, then, may well suggest that research in medical and engineering sciences preferentially locates in larger cities benefitting from the local presence of patients and companies engaged in related research. That is, these disciplines look for “institutional complementarities” [18]. While in some (big) sciences the most important complementarities take place with large experimental facilities, according to Bonaccorsi [19] in other fields, like engineering, medical sciences and life sciences they are most likely to take the form of human capital and institutional complementarities with both discovery and invention, requiring a structured interdependence between universities and local audiences (e.g. industry, hospitals).

Reversely, for some disciplines we observe a sublinear relationship between urban size and scientific production as indicated by *β<1*. For example, arts and humanities, decision sciences and veterinary sciences show low values. Typically, the statistical fit of the scaling law is also weakest for these disciplines, which makes it harder to draw conclusions from the beta coefficients. Nevertheless, the low value for veterinary sciences may not come as a surprise given, as one expects research in this area to be over-represented in more rural rather than urban areas.

We also find large differences between disciplines regarding the city size with maximum variance of residuals. Recall that this city size indicates the city size at which there are some exceptional “science cities” producing many more papers than what is expected from the power law. At the level of disciplines, these exceptional cities are not necessarily the typical universities located in small “campus towns” but also cities that host a specialized research institute dedicated to research in a particular disciplinary area (e.g., medical school or a business school).

Interestingly, the city size with maximum variance in scientific production differs greatly per discipline (see last column of Table 1, see more detail in Text S1 and Figure S1). The city size with the maximum estimated variance ranges from less than 150,000 inhabitants for agricultural and biological sciences, environmental science and social sciences, to over a 500,000 for most of the medical sciences (dentistry, nursing, health professions) and multidisciplinary sciences. The city size with maximum variance is indicative of a critical mass that would support the development of disciplinary research strength.

## Conclusions and Policy Reflections

The geography of scientific knowledge production is very uneven. For instance, the world’s most influential scientific researchers reside in a very small number of cities [20]. This is reinforced by research linkages which connect in particular the scientific hubs. At the same time, there is a process of ongoing globalization in scientific research [21], as illustrated by the ever increasing number of locations that contribute to scientific publications.

In this context, it seems obvious to focus on scaling patterns for policy making with respect to the scientific performance of cities given their city size. In this paper, we showed that scaling laws do not fully apply to the production of scientific knowledge. Indeed, the per capita number of scientific papers increases with city size and the exponent is very high, but the statistical regularity remains weak.

We also stressed that the overrepresentation of science in larger cities does not indicate in itself that scientific knowledge production benefits from locating in larger cities. The scaling between population and scientific output does not necessarily indicate that there are agglomeration advantages. Alternatively, these patterns may emerge from the preference of staff and students to live in larger cities, the mobility of productive scientists towards elite institutions in large cities, or a bias in the allocation of public funds towards larger cities.

Scaling laws could also be estimated on the basis of the urban population of scientists alone, rather than on the basis of the total urban population. Such data are currently not available, and difficult (but not impossible) to construct from publication data. It is most likely that the explanatory power of the scaling function would then be much higher. This issue can be taken up in future research.

However, this study does raise the additional question whether the specific type of research activity undertaken matters? This question is important because there are clear policy implications of this issue in terms of policies directed towards the concentration of innovation and knowledge development. The results show a great deal of heterogeneity across disciplines. While some disciplines (Arts and Humanities, Decision Sciences and Veterinary Sciences) show low scaling values, other disciplines show strong signs of scaling, in particular Medicine, Biochemistry, Genetics and Molecular Biology and Engineering.

What these disciplines have in common is that they are characterized by intensive interaction with local stakeholders, be it patients or companies in a local context of application. The high beta coefficients suggest that research in these fields preferentially locates in larger cities benefitting from the local presence of patients and companies engaged in related research. These patterns can be expected to be particularly relevant for new and divergent areas of knowledge production [22]. These areas are typically not based, like big science, on large physical infrastructures, but on local complementarities in the cognitive approach of scientists and in the institutional settings involved. According to Bonaccorsi [19] new sciences require above all the mobilization of cognitively heterogeneous teams and formalized collaboration between academia and other institutions, such as hospitals, government laboratories, regulatory agencies, or industry. In large cities, these requirements are more easily met.

As a consequence, especially large metropolitan areas can benefit from the diversity of human resources and institutional complementarities that provide comparative advantages to yield greater output in terms of knowledge production. The key policy concerns would then become how to identify the new fields of knowledge production and how to foster such diversity of human resources and institutional complementarities.

We also observed that there is a high variance in scientific output for mid-sized cities. For this class of cities, a few cities excel while most underperform. The exact maximum variance in scientific output is reached at different city sizes in different disciplines. In some disciplines (Agricultural and Biological Sciences, Environmental Science and Social Sciences) very small cities with less than 150,000 can already excel while for other disciplines (several Medical Sciences and Multidisciplinary Sciences), such high exceptional performance is only observed for city exceeding half a million inhabitants.

The city size with maximum variance is indicative of a critical mass that would support the development of disciplinary research strength. Hence, for small cities, a strategic investment in research that displays high variance at large city sizes (such as the Medical Sciences just mentioned as well as interdisciplinary research) entails a high risk. Reversely, for large cities, the opportunities to excel may be precisely in these sciences as little competition is expected from smaller cities. In particular, one may expect that diversified knowledge bases and various institutional complementarities may be important at the frontier of science, where new interdisciplinary directions of research emerge by bringing together diverse skills and diverse contexts of application. In these respects, large cities can be expected to have a comparative advantage.

## Supporting Information

Figure S1 **Scatter plots of the linear regression residuals (in absolute value) and the respective variance estimated as function of city size.** (A) All disciplines. (B) Arts and humanities. (C) Chemistry. (D) Engineering.
(TIF)

Text S1 **On the inverted U-shaped heteroskedasticity of the linear regression residuals.**
(DOCX)
