# Supplementary figures and images for: On Scaling of Scientific Knowledge Production in U.S. Metropolitan Areas
Source: PLoS One. 2014 Oct 29;9(10):e110805. doi: 10.1371/journal.pone.0110805 (PMC4212974; doi:10.1371/journal.pone.0110805)

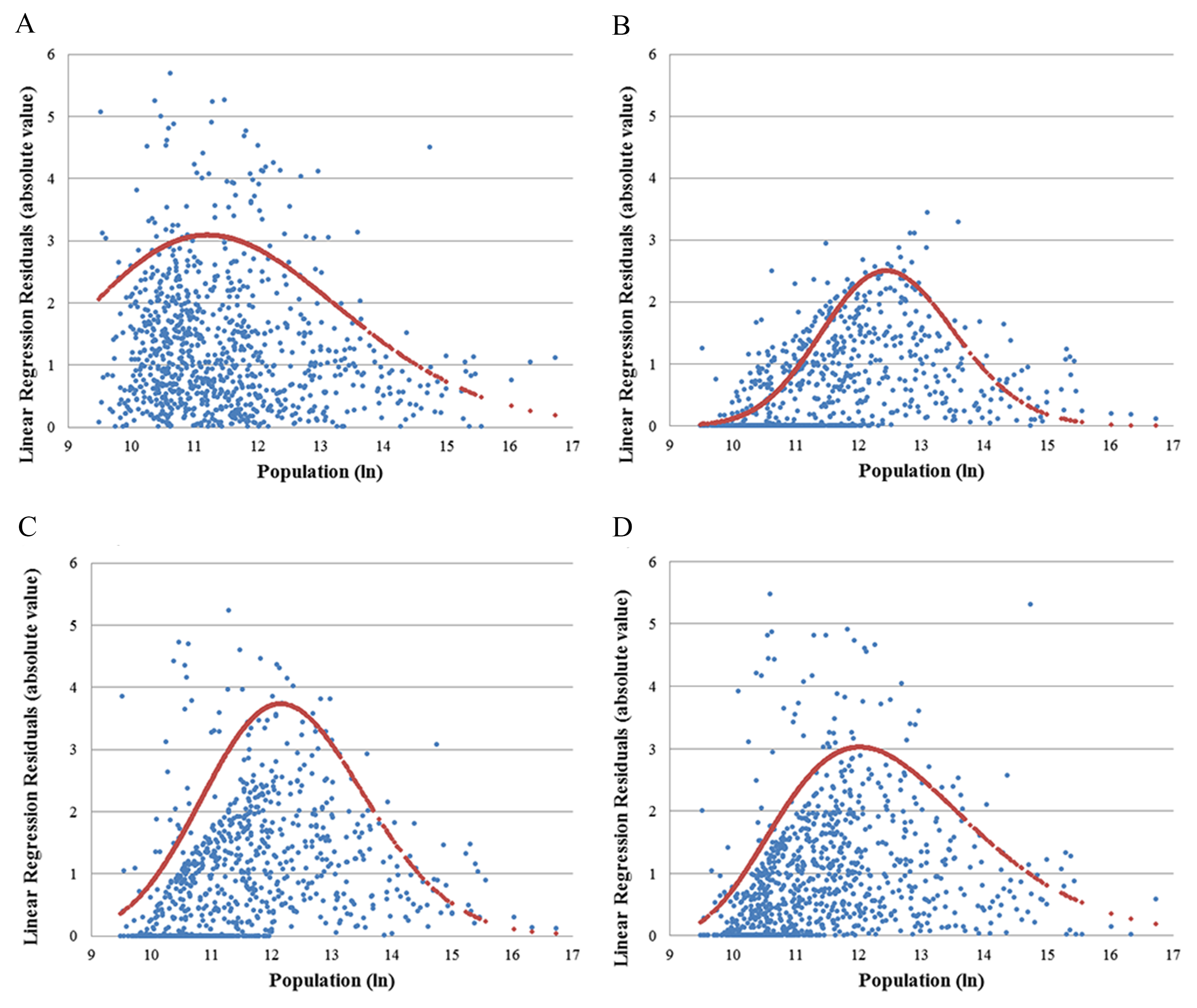

Supplement: Figure S1 — Scatter plots of the linear regression residuals (in absolute value) and the respective variance estimated as function of city size. (A) All disciplines. (B) Arts and humanities. (C) Chemistry. (D) Engineering. (TIF) [file pone.0110805.s001.tif]
