# Supplementary material for: On Scaling of Scientific Knowledge Production in U.S. Metropolitan Areas
Source: PLoS One. 2014 Oct 29;9(10):e110805. doi: 10.1371/journal.pone.0110805 (PMC4212974; doi:10.1371/journal.pone.0110805)
Supplement: Text S1 — On the inverted U-shaped heteroskedasticity of the linear regression residuals. (DOCX) [file pone.0110805.s002.docx]

**Supporting information**

For the dual purposes of correction for heteroskedasticity as well as a systematic exploration of the inversely U-shaped variation in regression residuals, we use one integral estimation method. In case of *a priori* information that can specify heteroskedasticity as some function $f(\mathrm{N}_{i})$ of the independent variable of the linear regression such that $\sigma_{i}^{2}=\sigma^{2}f^{2}(\mathrm{N}_{i})$,

one resorts to weighted least squares estimation where observation *i* is weighted by $1/f(\mathrm{N}_{i})$ for all *i*.

Nevertheless, since the inversely U-shaped heteroskedasticity we observe here is a new phenomenon, we have no such prior information except for what we can observe empirically. The skewed, bell-shaped pattern observed in Figure 1 is representative of all disciplines. Hence, we use one and the same functional form in all regressions.

The functional form that can best capture this pattern is the probability distribution function of the Gamma distribution$\Gamma(\mathrm{N}_{i},\theta,\gamma)$, where *θ* and *γ* are parameters. Accordingly we assume $\sigma_{i}^{j}=\sigma^{j}\delta^{j}\Gamma(\mathrm{N}_{i},\theta^{j},\gamma^{j})$and estimate the parameters$\theta^{j}$, $\gamma^{j}$ as well as $\delta^{j}$ (which is yet another parameter that scales the functional form) and by ordinary least squares estimation on the basis of the residuals of initial (non-weighted) linear regression estimation. Note that the superscript *j* denotes individual disciplines.

Figure S1 depicts the estimated variance functions (i.e., the continuous red curves) along with the scatter plot of absolute values of the respective error terms against (log) city size for the whole dataset (all disciplines together) and three selected disciplines. These examples also show that, despite the similarity in the general form, scientific disciplines are heterogeneous in terms of the variation not explainable by scaling laws. For example, while such variance is highest at the city size of 73,466 (i.e., *e^11.205^*) at the aggregate level, it is 252,760 for arts and humanities and 165,703 for engineering.
